# Regulated cell death in glioma: promising targets for natural small-molecule compounds

**DOI:** 10.3389/fonc.2024.1273841

**Published:** 2024-01-18

**Authors:** Mingyu Han, Sui Li, Huali Fan, Junsha An, Cheng Peng, Fu Peng

**Affiliations:** ^1^West China School of Pharmacy, Sichuan University, Chengdu, China; ^2^State Key Laboratory of Southwestern Chinese Medicine Resources, Chengdu University of Traditional Chinese Medicine, Chengdu, China; ^3^Key Laboratory of Drug-Targeting and Drug Delivery System of the Education Ministry, Sichuan Engineering Laboratory for Plant-Sourced Drug and Sichuan Research Center for Drug Precision Industrial Technology, Sichuan University, Chengdu, China

**Keywords:** regulated cell death, glioma, natural small-molecule compounds, regulatory factors, pathways

## Abstract

Gliomas are prevalent malignant tumors in adults, which can be categorized as either localized or diffuse gliomas. Glioblastoma is the most aggressive and deadliest form of glioma. Currently, there is no complete cure, and the median survival time is less than one year. The main mechanism of regulated cell death involves organisms coordinating the elimination of damaged cells at risk of tumor transformation or cells hijacked by microorganisms for pathogen replication. This process includes apoptosis, necroptosis, autophagy, ferroptosis, pyroptosis, necrosis, parthanayosis, entosis, lysosome-dependent death, NETosis, oxiptosis, alkaliptosis, and disulfidaptosis. The main goal of clinical oncology is to develop therapies that promote the effective elimination of cancer cells by regulating cell death are the main goal of clinical oncology. Recently, scientists have utilized pertinent regulatory factors and natural small-molecule compounds to induce regulated cell death for the treatment of gliomas. By analyzing the PubMed and Web of Science databases, this paper reviews the research progress on the regulation of cell death and the role of natural small-molecule compounds in glioma. The aim is to provide help for the treatment of glioblastoma.

## 1 Introduction

The most prevalent malignant primary brain tumor in adults is glioma, which is typically categorized into limited glioma, which is benign type that can be cured through complete surgical excision, and diffuse glioma, which is more malignant and incurable after surgical excision alone. According to the 2021 World Health Organization classification, gliomas are categorized into four grades (1–4) (1) (**Table 1**). High grade glioma includes anaplastic astrocytoma, anaplastic oligodendroglioma and glioblastoma (GBM), which are classified as grade 3 to 4 by WHO. Unfortunately, more than 50% of glioma patients progress to the most malignant form of glioma, GBM with a median survival time of less than 8 months (2). Gliomas have many causes, mainly classified as environmental factors and cellular control at the cell level. Environmental factors, include exposure to therapeutic ionizing radiation, as well as exposure to substances such as vinyl chloride or pesticides, smoking, petroleum refining or production work, and employment in the synthetic rubber manufacturing industry (3). Numerous uncontrolled cellular processes exist, such as cell cycle regulation, growth factor expression, angiogenesis, invasion, migration barriers, genetic instability, and apoptosis (4). The complexity of GBM is evident at the cellular and genomic levels, and they exhibits significant inter- and intra-tumor heterogeneity (5). The standard treatment for glioma primarily involves surgical resection, along with radiotherapy, chemotherapy, and other comprehensive therapies. The most common approach worldwide is the combination of temozolomide and radiation therapy, which has become the standard treatment for adults newly diagnosed with GBM (6). However, this treatment remains ineffective. Interestingly, immunotherapy, targeted therapy (6), and electric field therapy have been investigated recently in preclinical studies, suggesting a promising approach in the treatment of glioma. Cell proliferation and apoptosis are balanced in healthy organisms, but in disease conditions such as tumors, cell proliferation exceeds apoptosis (7). The primary objective of clinical oncology has been to develop therapies that facilitate the effective elimination of cancer cells through regulated cell death. In recent years, research has continued to identify various mechanisms of programmed cell death.

**Table 1:** Classification of different glioma grades.

Tumor	Classification	Grade	IDH	Level	Reference
Oligodendroglioma	1p/19q codeleted/coded or TERT promoter Oligodendroglioma; Anaplastic features or high mitotic index or CDKN2A/B homozygous deletion Oligodendroglioma	(1) Grade 2; (2) Grade 3	(1) IDH mutant; (2) IDH mutant	(1) Low-grade glioma; (2) High-grade glioma	(1)
Astrocytoma	(1) ATRX loss or TP53 mutation astrocytoma; (2) ATRX loss or TP53 mutation or anaplastic features or high mitotic index astrocytoma; ATRX loss or TP53 mutation or CDKN2A/2B homozygous deletion or CDKN2A/B astrocytom; Pilocytic astrocytoma	(1) Grade 2; (2) Grade 3; (3) Grade4; (4) Grade 1	(1) IDH mutant; (2) IDH mutant; (3) IDH mutant	(1) Low-grade glioma; (2) High-grade glioma; (3) High-grade glioma; (4) Low-grade glioma	(1)
Diffuse hemisheric glioma	H3.3 G34R/V-mutant diffuse hemisheric glioma; H3 K27M-mutant or loss of H3K27me3 diffuse midline glioma	Grade 4	IDH mutant	High-grade glioma	(1)
Glioblastoma	Chromosome 7 gain or chromosome 10 loss or homozygous loss CDKN2A/2B or TERT promoter mutation or ATRX retained or EGFR amplification glioblastoma	Grade 4	IDH wild	High-grade glioma	(1)

**Figure 1:**
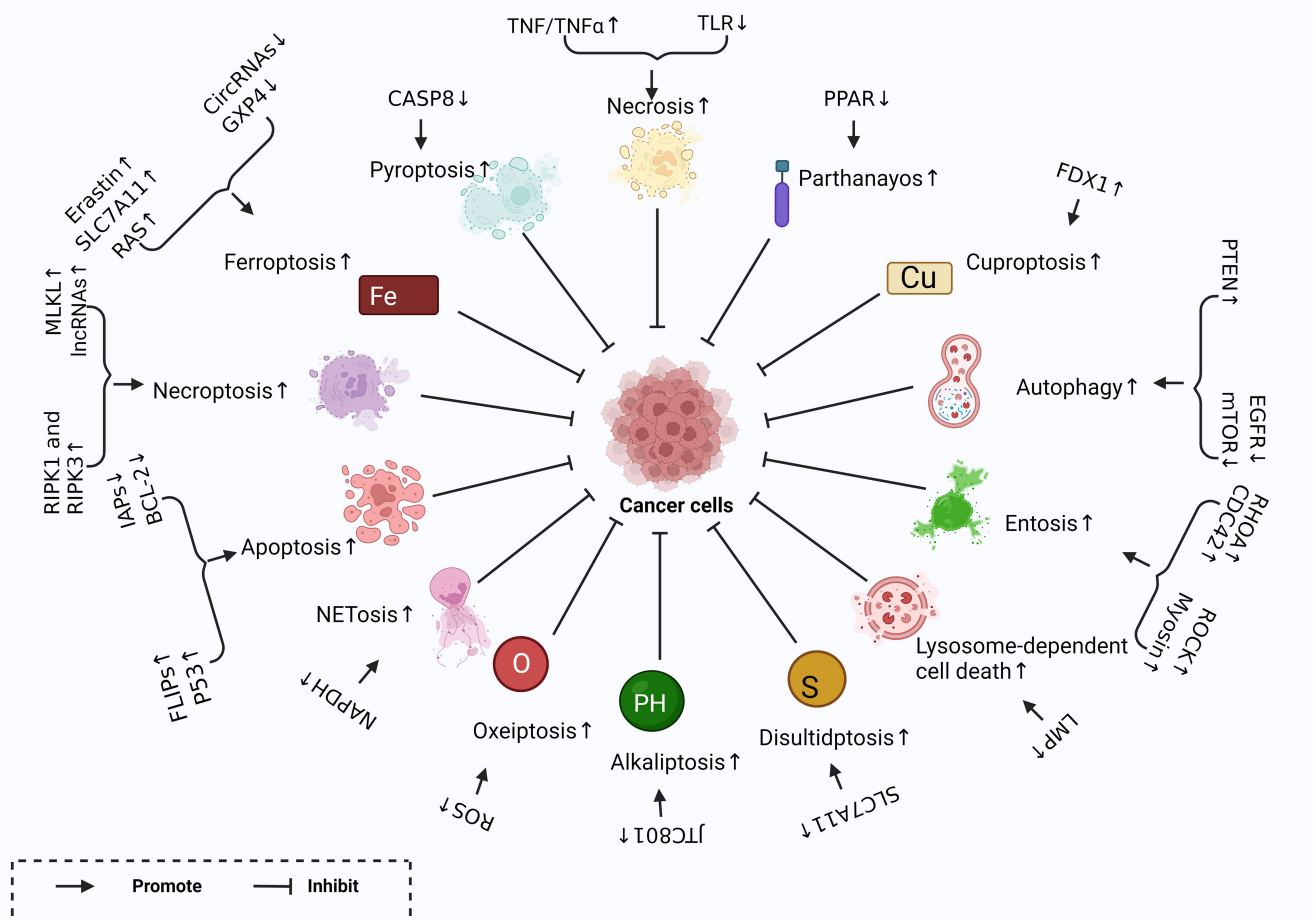
Different Forms of RCD and related regulatory factors created with BioRender.com. Different regulatory factors inhibit tumor growth by up-regulating or down-regulating different ways of cell regulatory death.

**Figure 2:**
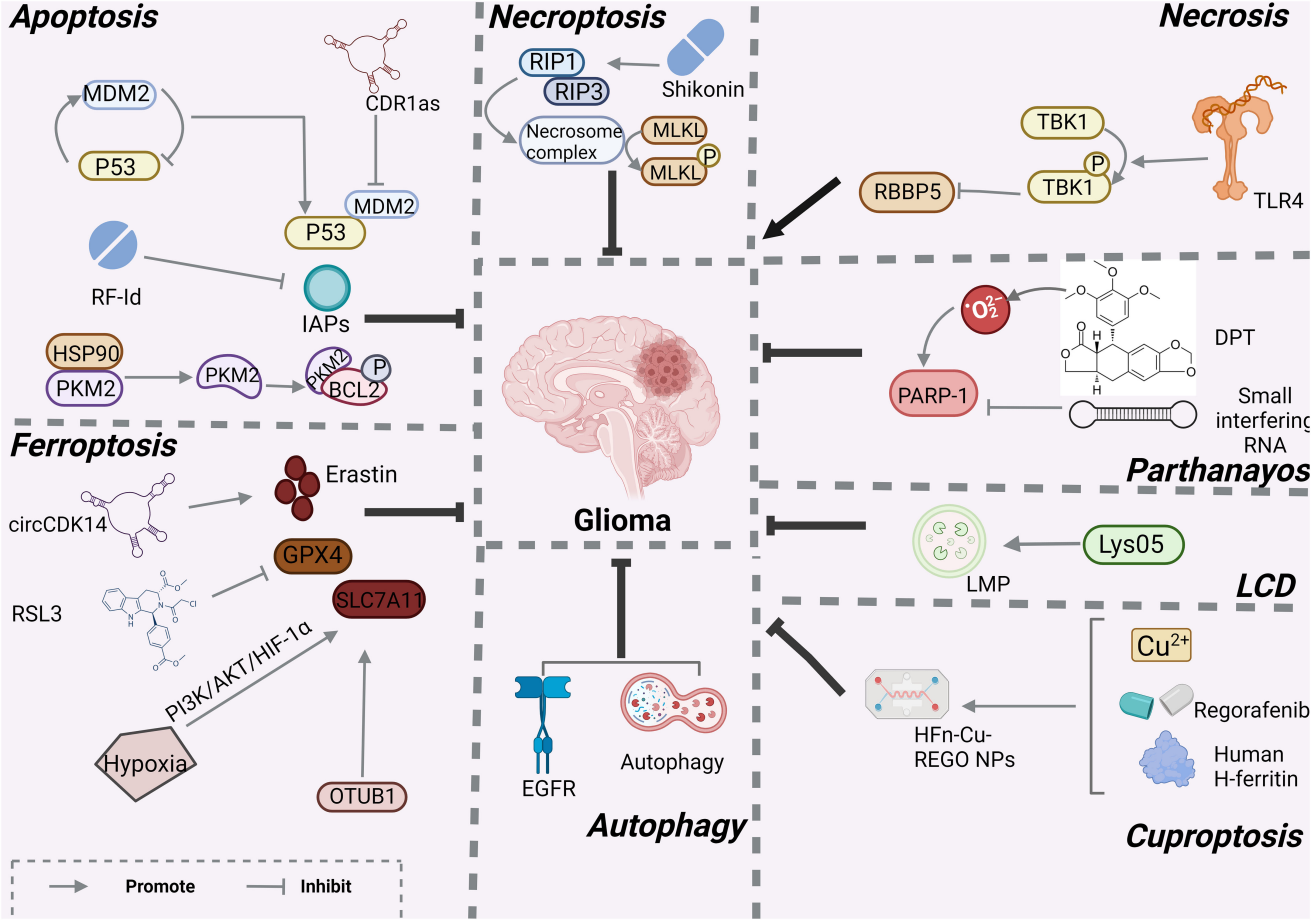
Mechanisms associated with RCD in glioma created with BioRender.com. At present, only apotosis, necroptosis, necrosis, LCD, ferroptosis, autophagy and cuprotosis have been studied in gliomas, and other programmed modes of death have not been demonstrated.

Over the past decades, apoptosis has been extensively studied as an important cancer defense mechanism and has been used in the development of targeted anti-cancer drugs. However, because cancer cells have endogenous or acquired apoptotic resistance, the therapeutic effect of related drugs is not ideal, so it is necessary to explore non-apoptotic cell death pathways that can be used to kill drug-resistant cancer cells. Regulated cell death (RCD) during development or tissue renewal, depends on specific molecular mechanisms and is regulated. Many of the fundamental processes include organogenesis and tissue remodeling, the removal of unnecessary structures or cells, and the regulation of cell numbers, which are facilitated by is done with the participation of RCD. Moreover, it is the primary mechanism through by which organisms eliminate damaged cells at risk of tumor formation or cells hijacked by microorganisms for pathogen replication. This process can be induced by developmental programs and stress-induced signals that stimulate membrane-bound and cytoplasmic proteins through a complex cascade of transcriptional changes and post-translational protein modifications (8). RCD, particularly apoptosis and necroptosis, serves as a natural barrier that restricts the survival and dissemination of malignant cells. However, cancer cells have evolved various strategies to evade this process by generating genetic mutations or epigenetic modifications in key regulators of the pathway (9). RCD has introduced new approaches and strategies for cancer treatment. Drugs and other interventions can inhibit the cancer invasion, migration, and spread by targeting RCD, or they can cancer cell death (**Figure 1**). The types of RCD for glioma can be mainly divided into the following two categories. One type has made research progress in the treatment of glioma and this review will also introduce these findings in detail, such as apoptosis, necroptosis, autophagy, ferroptosis, pyroptosis, parthanayos, entosis, lysosome-dependent cell death and cuproptosis (**Table 2**); the other may play the potential role for glioma treatment in furfure, for example, entosis (109, 110), NETosis (102, 103), oxeiptosis (111), alkaliptosis (112, 113) and disulfidptosis (114) (**Figure 2**).

**Table 2:** The types of RCD and their related morphology, immunological characteristics and regulating factors.

Classification	Morphology	Immunological characteristic	Regulating factors	Reference
Apoptosis	Nuclear chromatin condensation and fragmentation, endoplasmic reticulum expansion, organelle retention, and cytoplasmic contraction	ICD	(1) Inhibitor of apoptosis: P53、FLIPs、IAPs, et al. (2)Bcl-2 family.	(10–28)
Necroptosis	Cell swelling, supracellular membrane pore formation, plasma membrane rupture and moderate chromatin condensation	ICD	(1) PRK1 and RIPK3 (2) lncRNA (3) MLKL	(29–37)
Autophagy	The Golgi apparatus and other organelles swell, the nucleus solidifies, a large number of phagocytic vesicles are formed, and the cytoplasmic membrane undergoes specialization	ICD	PTEN; EGFR; ATG family; Beclin1and mTOR, et al.	(38–47, 48–56)
Ferroptosis	Mitochondrial cristae reduction (disappearance), mitochondrial outer membrane rupture, wrinkling, dark mitochondrial color, iron-dependent nucleus without rupture and cell membrane rupture	ICD	Erastin; RSL3; RAS; FSP1; SLC7A11; NRF2; Ferrostatin-1; Liproxstatin-1, et al.	(55, 57–74)
Pyroptosis	Cells continue to swell until the cell membrane ruptures	ICD	Cystathione aspartase -1/2/3/4/5/6/7/8/9/10/11	(75–79)
Necrosis	Cell distension, rupture of the cell membrane, spillage of cell contents, slower nuclear changes, inadequate DNA degradation, causing severe local inflammatory reactions	ICD	TNF receptor superfamily; T cell receptor; Interferon receptor; Toll-like receptor (TLR), et al.	(80–96)
Parthanayos	Chromosome condensation, DNA fragmentation	ICD	PARP1; PARG; ARH3; AIF; MIF, et al.	(97–99)
Lysosome-dependent cell death	Lysosomal rupture	ICD	Cathepsins; STAT3; TP53; NF-κB; MCOLN1, et al.	(100, 101)
NETosis	Chromatin deprotonation, nuclear membrane disruption and release of chromatin fibers	ICD	ELANE; MMP; MPO; CAMP/LL37; PADI4, et al.	(102–104)
Cuproptosis	Similar to Ferroptosis.	ICD	FDX1; Protein lipid acylation; DLAT; LIAS; pyruvate; alpha-ketoglutarate; HSP70, etc.	(105–108)
Apoptosis	Nuclear chromatin condensation and fragmentation, endoplasmic reticulum expansion, organelle retention, and cytoplasmic contraction	ICD	(2) Inhibitor of apoptosis: P53、FLIPs、IAPs, et al.(2) Bcl-2 family.	(10–28)
Necroptosis	Cell swelling, supracellular membrane pore formation, plasma membrane rupture and moderate chromatin condensation	ICD	(2) PRK1 and RIPK3 (2) lncRNA (3) MLKL	(29–35, 37)
Autophagy	The Golgi apparatus and other organelles swell, the nucleus solidifies, a large number of phagocytic vesicles are formed, and the cytoplasmic membrane undergoes specialization	ICD	PTEN; EGFR; ATG family; Beclin1and mTOR, et al.	(38–56)
Ferroptosis	Mitochondrial cristae reduction (disappearance), mitochondrial outer membrane rupture, wrinkling, dark mitochondrial color, iron-dependent nucleus without rupture and cell membrane rupture	ICD	Erastin; RSL3; RAS; FSP1; SLC7A11; NRF2; Ferrostatin-1; Liproxstatin-1, et al.	(55, 57–74)
Pyroptosis	Cells continue to swell until the cell membrane ruptures	ICD	Cystathione aspartase -1/2/3/4/5/6/7/8/9/10/11	(75–79)
Necrosis	Cell distension, rupture of the cell membrane, spillage of cell contents, slower nuclear changes, inadequate DNA degradation, causing severe local inflammatory reactions	ICD	TNF receptor superfamily; T cell receptor; Interferon receptor; Toll-like receptor (TLR), et al.	(80–96)
Parthanayos	Chromosome condensation, DNA fragmentation	ICD	PARP1; PARG; ARH3; AIF; MIF, et al.	(97–99)
Lysosome-dependent cell death	Lysosomal rupture	ICD	Cathepsins; STAT3; TP53; NF-κB; MCOLN1, et al.	(100, 101)
NETosis	Chromatin deprotonation, nuclear membrane disruption and release of chromatin fibers	ICD	ELANE; MMP; MPO; CAMP/LL37; PADI4, et al.	(102–104)
Cuproptosis	Similar to Ferroptosis.	ICD	FDX1; Protein lipid acylation; DLAT; LIAS; pyruvate; alpha-ketoglutarate; HSP70, etc.	(105–108)

**Figure 3:**
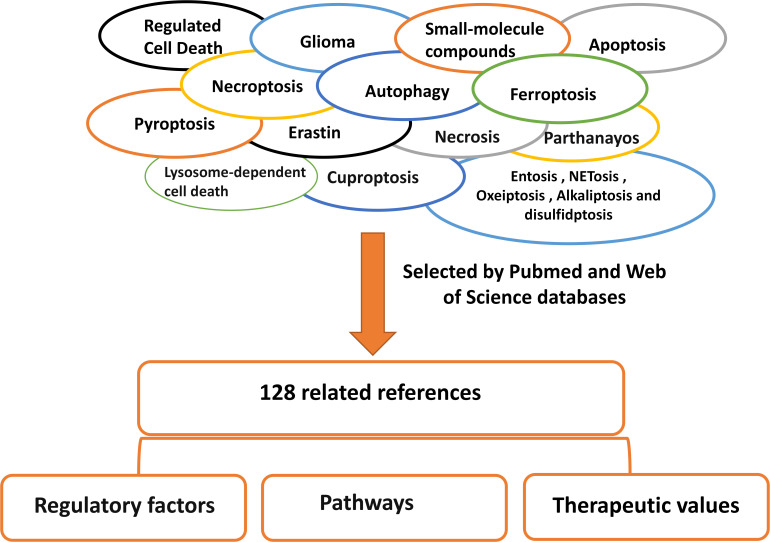
Literature search method/strategies (databases and keywords involved). We searched and screened 138 articles through Pubmed and web of science for classification and summary, and reported the research of RCD in glioma.

Small-molecule compounds are organic compounds with molecular weights of less than 900 Da. Numerous studies have conducted phenotypic, genomic, epigenomic, transcriptomic, and proteomic analyses, utilized disease models to explore potential inhibitors, and evaluated candidate small-molecule drugs in clinical settings (115). Simultaneously targeting of small-molecule compounds has become an effective approach for treating cancer (116). For instance, in the management of triple-negative breast cancer, DOT1L is crucial for developing an aggressive phenotype by promoting EMT/CSC through its interaction with c-Myc and p300 acetyltransferases (117). Natural small-molecule compounds are animal, plant, and mineral-derived drugs that have been recognized by modern medical systems for their specific pharmacological activities. Plants have been utilized as medicinal remedies for at least 60,000 years due to their capacity to generate combinations of secondary metabolites with a broad spectrum of pharmacological properties, including anti-cancer effects (118). Currently, many combination drugs, such as statins, are not suitable for treating malignant gliomas (119). Therefore, the study of natural drugs is particularly important. Herbs have deep roots in many cultures and traditions, and according to a study, and one in three cancer survivors reports uses them (120). Natural small-molecule compounds have been widely extensively utilized as drugs or adjuvant chemotherapy agents in cancer treatment due to their selective ability to kill cancer cells, reduce drug resistance, and alleviate side effects (121). At the same time, compounds derived from plants have been shown to play a role in many cancers. For example, lycopene, a carotenoid found in many fruits, and resveratrol have been shown to play a role in breast and oral cancers (122). Many natural small molecule compounds have been studied and validated to play a role in GBM and can be categorized mainly from marine organisms [e.g., antitumour (123)], proteins (e.g., carnosine [β-alanyl-L-histidine) (124)], and plants [e.g., eucalyptal A (125), galbanic acid (126), gossypol (127), rupesin E (127), Tectorigenin (128) and Withaferin A (129)], and also co-administered (e.g. berberine and solid lipid curcumin particles (130), propolis and bacopa monnieri (L.) wettst. (Brahmi) (131)). By analyzing PubMed and Web of Science databases, this paper reviews the research progress on the regulation of cell death and the role of natural small-molecule compounds in glioma, hoping to provide help for the treatment of glioma (**Figure 3**).

## 2 Different forms of RCD involved in glioma

### 2.1 Apoptosis

Apoptosis is the process by which cells automatically terminate their lives under certain physiological or pathological conditions, and it is controlled by intrinsic genetic mechanisms. Apoptosis eliminates redundant cells, non-functional cells, cells that have developed abnormally, or harmful cells. The key characteristics of apoptosis include the condensation and fragmentation of nuclear chromatin, expansion of the endoplasmic reticulum, retention of organelles, and cytoplasmic shrinkage. The treatment of glioma patients involves promoting the apoptosis of cancer cells, thereby inhibiting the proliferation of glioma cells. Apoptosis is initiated by apoptotic signals, followed by the interaction of apoptosis regulatory molecules to activate protein hydrolases (caspases), leading to a continuous cascade of reactions. Numerous molecules regulate apoptosis, including the following: (1) apoptosis inhibitory molecules such as P35, FLIPs, and Inhibitors of Apoptosis (IAPs); and (2) the Bcl-2 family which includes Mcl-1, NR-B, A1, Bcl-w, and Bcl-x.

#### 2.1.1 P53

Mutation and/or inactivation of the tumor suppressor P53 is essential for tumorigenesis. Malignant tumors often have mutations in TP53, leading to the development of an oncogenic phenotype. Inactivation of P53 is crucial for the development of gliomas, particularly GBM (10). Furthermore, MDM2 plays a significant role as a negative regulator of P53 by binding to P53 and forming a stable complex to regulate its activity. A study by Jiacheng Lou et al. demonstrated that the cyclic RNA CDR1as disrupts the P53/MDM2 complex to inhibit GBM (11). In addition, prostate apoptosis response-4 regulates apoptosis to induce GBM cell death by upregulating p53 and BNIP3 (28).

#### 2.1.2 Fas-associated death domain (FADD)-like IL-1β-converting enzyme-inhibitory proteins (FLIPs)

FLIPs, known for its role in cystathione-8, are multifunctional proteins that play a role in regulating important cellular processes that includes apoptosis, necroptosis, autophagy, inflammation, innate immunity (12) adaptive immunity, and embryonic development (13). FLIP plays a role in numerous pathways, including the Wnt (14), NFκB (15), and mitogen-activated protein kinase (MAPK) pathways (16), et al. A study found that targeting Karyopherin β1 (KPNβ1) can overcome tumor necrosis factor-related apoptosis-inducing ligand (TRAIL) resistance by modulating FLIP in GBM cells. The study also suggested that this combination therapy may soon enter clinical trials for anti-cancer treatment (17).

#### 2.1.3 IAPs

IAPs are often overexpressed in cancer cells, where they inhibit caspase activation and apoptosis, ultimately leading to drug resistance (18). In addition to regulating RCD, IAPs also control MAPK and both typical and atypical NF-κB pathways, which in turn leads to the transcription of target genes. Previous studies have found that the synthesis of the compound Derivative 3-[(decahydronaphthalen-6-yl)methyl]-2,5-dihydroxycyclohexa-2,5-diene-1,4-dione (RF-Id) inhibits IAP family proteins and the NFκB pathway, inducing apoptosis in GBM cells. This makes it a promising lead compound for the development of a new class of anti-cancer drugs with multiple targets (19).

#### 2.1.4 B-cell lymphoma 2 (Bcl2) family

Bcl2 is an anti-apoptotic member of the Bcl-2 family that isupregulated in many human cancers. Multiple members of the Bcl-2 family of apoptosis-regulating proteins include six anti-apoptotic agents, three structurally similar pro-apoptotic proteins, and several structurally varying pro-apoptotic interacting proteins that act as upstream agonists or antagonists (20). The Bcl-2 family acts in mitochondria, endoplasmic reticulum, and lysosomes, and is the biological “guardian” of cellular organelles. However, members of the Bcl-2 family are not effective in treating tumor cells (21). The pyruvate kinase M2 isoform (PKM2) phosphorylates Bcl2 and directly inhibits apoptosis, demonstrating that the HSP90-PKM2-Bcl2 axis is a potential target for therapeutic intervention in GBM (22). In addition, the combination of ABT-263 and MLN8237 reduced mitotic slippage and polyploidy and promoted the elimination of mitosis-deficient cells through the BAX/BAK-dependent cysteinase-mediated apoptotic pathway. WEHI-539, a B-cell lymphoma extra-large (BCL-XL) antagonist, significantly enhanced tumor cell killing when combined with MLN8237 and resistant brain tumor cells sensitive to the novel BAX activator SMBA1. In addition, siRNA-mediated Bcl-xL sensitizes pGBM and medulloblastoma cells to MLN8237 replicates the beneficial effects of combined drug treatments (23).

Recent studies have shown that the action of regulatory factors such as P53, FLIPs, IAPs, and members of the Bcl-2 family members may promote apoptosis through other regulatory factors, pathways, enzymes, or increased drug sensitivity in cancer cells. This provides more opportunities more possibilities for anti-cancer experiments.

### 2.2 Necroptosis

Necroptosis is a programmed lytic cell death pathway believed to be involved in eliminating pathogen-infected and/or damaged cells in specific degenerative or inflammatory diseases. It shares key morphological features with apoptosis, leading to cell swelling, formation of pores in the suprabasal layer cell membrane, rupture of the plasma membrane, and moderate chromatin condensation. However, the precise role of necroptosis in glioma remains unclear. Numerous regulators of necrotic cell death exist, including PRK1, RIPK3, long non-coding RNA (lncRNAs), and mixed lineage kinase domain-like pseudokinase (MLKL), among others.

#### 2.2.1 RIPK1 and RIPK3

PRK1 and RIPK3 can assemble as oligomeric complexes known as necrosomes, and necroptosis can occur downstream them (29). In a cohort of over 60 cancer cell lines, it was observed that two-thirds of the RIPK3 protein levels were reduced, suggesting that necroptosis can effectively inhibit tumor growth. Furthermore, low expression of RIPK3 was found in patients with multiple cancers with poor prognosis (30). Among the seven prognostic necroptosis genes, especially RIPK1, RIPK3, FAS and FADD, can construct risk profiles and predict the prognosis of glioma patients. The role of this risk profile can be related to the immune response and angiogenesis, enhancing inflammatory activity and attracting immunosuppressive cell infiltration to form a chronic inflammatory microenvironment, which promotes GBM growth (37).

#### 2.2.2 LncRNA

LncRNAs, as a class of RNA molecules with transcriptional lengths greater than 200 nucleotides, do not encode proteins but are involved in protein-coded gene regulation in the form of RNA. lncRNAs play important roles in dosage compensation, epigenetic regulation, cell cycle regulation, and cell differentiation regulation (31). Some scientists have found predictive models for 12 lncRNAs to help assess glioma patients’ prognosis and molecular characteristics. For example, GSEA-GO is associated with Wnt, inositol phosphate metabolism, butyrate metabolism, long-term depression, and taste transduction, improving treatment modality and potentially stimulating future exploration of glioma formation and treatment (32). Search for specific biomarkers based on markers of necroptosis may be more precise and effective in LGG and GBM management, which may provide new clues for immunotherapy and prognosis.

#### 2.2.3 MLKL

MLKL undergoes rapid membrane permeabilization, thereby mediating the release of intracellular contents, including immunogenic Danger-Associated Molecular Patterns. Necroptosis is thought to be a self-sacrificing strategy for tumor proliferation and metastasis (33). MLKL promotes shikonin-induced necroptosis in glioma cells by promoting chromatin lysis, and shikonin induces positive feedback between MLKL and its upstream signals RIP1 and RIP3, thereby promoting the death of glioma including GBM cells (34).

The exact role of necroptosis in gliomas remains relatively unknown, and the prognostic effect of necroptosis on gliomas is still not particularly obvious according to current algorithms.

### 2.3 Autophagy

Autophagy (38) is a process in which damaged proteins or organelles are enclosed in autophagic vesicles with a bilayer membrane structure and then transported to lysosomes (in animals) or vacuoles (in yeast and plants) for degradation and recycling. Electron microscopy revealed that organelles such as the Golgi apparatus had swollen, the nucleus had solidified, numerous phagocytic vesicles had formed, and the cytoplasmic membrane had undergone specialization. Cellular autophagy can be categorized into macroautophagy, microautophagy, and molecular chaperone-mediated autophagy (CMA). Autophagy occurs in five stages: (1) phagocytosis or nucleation. (2) conjugation of the ATG5-ATG12 complex, its interaction with ATG16L, and multimerization of the phagocytic vacuole, (3) processing of LC3 and its insertion into the swollen phagocytic mass membrane, (4) capture of selected targets for degradation, and (5) fusion of the autophagosome with the lysosome, followed by proteolytic degradation by the lysosomal protease contained in the absorbed molecule undergoes proteolytic degradation. The relationship between cellular autophagy and tumors is more complex. On the one hand, normal cells with increased autophagy can inhibit tumorigenesis. On the other hand, tumor cells can enhance their stress response, including autophagy, to counter hypoxia, metabolite and therapeutic agent induction. This response is influenced by nutrient availability, microenvironmental stress, pathogenic conditions, and the presence of the immune system (39, 40). Therefore, the inhibition of tumor cell growth through autophagy needs to be analyzed on a case-by-case basis. The regulatory factors that have been found to be associated with glioma include Phosphatase and Tensin Homolog (PTEN), Epidermal growth factor receptor (EGFR), ATG family, Beclin1, and Mammalian target of rapamycin (mTOR).

#### 2.3.1 PTEN

PTEN, a tumor suppressor frequently mutated in human cancers with multiple cytoplasmic and nuclear functions, has been identified as a tumor suppressor gene in various cancers with frequent deletions on human chromosome 10q23 (41). PTEN inactivation leads to the constitutive activation of the PI3K-AKT pathway, resulting in increased protein synthesis, cell cycle progression, migration, and survival (42). A drug screening of GBM stem cells (GSC) revealed proteasome inhibition as a targeted therapy. Proteasome inhibition specifically induces cell death in three-dimensional (3D) PTEN-deficient GBM-like organs and inhibits *in situ* tumor growth in murine PTEN-null GSC (43).

#### 2.3.2 EGFR

EGFR, a member of the tyrosine kinase receptor (RTK) family, regulates the development and maintenance of epithelial tissue. It is typically a constitutively active receptor that does not depend on ligands for its activity. This altered transport and downregulation lead to abnormal downstream signaling, thereby promoting tumor development (44). The combination of EGFR and autophagy regulation impairs cell migration and enhances the radiosensitivity of GBM, thereby improving treatment outcomes in patients with gliomas (45).

#### 2.3.3 mTOR

As a serine/threonine kinase, mTOR is a major regulator of cellular metabolism that promotes cell growth in response to environmental signals (46). mTOR exists in two distinct signaling complexes, mTORC1 and mTORC2: (1) mTORC1 integrates nutrient and growth factor signals to facilitate anabolic pathways like protein and lipid synthesis, while also inhibiting catabolic pathways such as lysosomal biogenesis and autophagy; (2) mTORC2 regulates cell survival, metabolism, and cytoskeletal organization through AGC family kinases. Given its role as a significant regulator of cellular metabolism and autophagy, mTORC1 has emerged as an appealing target for pharmacological manipulation of autophagy. Recently, a summary of several mTOR inhibitors and their applications for inducing autophagy in preclinical studies have been summarized (as presented in **Table 3**).

**Table 3:** Small-molecule compounds of mTOR inhibitors to induce autophagy and related pathways.

Small-molecule compounds	Pathways	Reference
Celastrol	ROS / JNK and Akt / mTOR	(49)
Sino-wcj-33 (SW33)	PI3K/AKT/mTOR and AMPK/mTOR	(50)
Xanthatin	PI3K-Akt-mTOR	(47)
Sempervirine (SPV)	Akt/mTOR	(51)
Flavokawain B	ATF4-DDIT3-TRIB3-AKT-MTOR-RPS6KB1	(52)
Amentoflavone (AF)	AMPK/mTOR	(53)
Prucalopride	AKT-mTOR	(54)
Triptolide	ROS/JNK and Akt/mTOR	(55)
β-asarone	P53/Bcl-2/Bclin-1 and P53/AMPK/mTOR	(56)
Arctigenin	AKT/mTOR	(48)

The relationship between autophagy and tumors is unique and has two sides. Therefore, the inhibition of tumor cell growth through cellular autophagy also needs to be continuously analyzed based on the specific circumstances. Numerous studies have shown that the role of autophagy in tumors can be effectively leveraged, and several regulatory factors, such as PTEN and EGFR, can not only inhibit growth but also enhance tumor sensitivity to drugs, offering new avenues for future glioma treatment.

### 2.4 Ferroptosis

Ferroptosis is a regulated form of cell death characterized by the iron-dependent accumulation of lipid peroxidation to lethal levels. When cellular cysteine transport proteins are inhibited (e.g., erastin), intracellular glutathione (GSH) is depleted, which eventually leads to the inactivation of glutathione peroxidase (GPX4). This leads to the buildup of lipid peroxidation to a level that triggers cell death, which can also be directly caused by the inhibition of GPX4 enzymes (e.g., GSH peroxidase 4 inhibitors like RSL3) (57). The morphological features of ferroptosis are quite specific, including a reduction (disappearance) of the mitochondrial cristae, rupture of the outer mitochondrial membrane, wrinkling, darkening of the mitochondria, iron-dependent nuclei without rupture, and cell membrane rupture. Ferroptosis can inhibit tumor growth in several ways. For example, it sensitizes GBM to Anti-PD1/L1 immunotherapy and promotes M2 to M1 polarization (70). Numerous factors regulate ferroptosis, including Erastin, RSL3, RAS, FSP1, circular RNAs (circRNAs), SLC7A11, NRF2, GPX4, Ferrostatin-1, and Liproxstatin-1.

#### 2.4.1 Erastin

Erastin, the first ferroptosis activator identified in 2003, was found to play a significant role. Erastin, the first ferroptosis activator identified in 2003, exhibits significant lethality in human tumor cells carrying mutations in the HRAS, KRAS, and BRAF oncogenes (58). Chen et al. utilized transmission electron microscopy to analyze the sensitivity of circCDK14 to erastin-induced ferroptosis in glioma cells. They also conducted mitochondrial, iron, and reactive oxygen species assays. The study revealed that overexpression and elevated levels of circCDK14 in glioma tissues and cell lines were associated with a poor prognosis in glioma, including GBM patients (59).

#### 2.4.2 RAS and RSL3

The most commonly mutated gene families in cancer include the RAS gene family (KRAS, NRAS, and HRAS) (60). The direct inhibition of mutant RAS by allele-specific inhibitors is the best therapeutic approach. Therapies targeting the RAS activation pathway or RAS effector pathway can be combined with direct RAS inhibitors, immune checkpoint inhibitors, or T cell-targeting approaches to treat RAS-mutant tumors. RAS-selective lethal 3 (RSL3) is a widely recognized inhibitor of GPX4 that triggers ferroptosis by suppressing GPX4 expression (61, 62). A study found that RSL3 induces ferroptosis by activating the NF-κB pathway and depleting GPX4 in GBM (63).

#### 2.4.3 SLC7A11

SLC7A11 is a cysteine/glutamate antiporter protein that synthesizes GSH and neutralizes oxidized substances in cell membranes (64). It was recently discovered to play an anti-cancer role in various cancers, including malignant glioma, by inhibiting SLC7A11-activated ferroptosis (65). In glioma, hypoxia upregulates SLC7A11 via the PI3K/AKT/HIF-1α axis to enhance glioma resistance to salazosulfapyridine-induced ferroptosis (66). Zhao et al. found that the ubiquitin hydrolase OTUB1 inhibits ferroptosis by stabilizing the SLC7A11 protein and promoting glioma cell stemness (67).

#### 2.4.4 Circular RNA

As a class of non-coding RNAs, circular RNAs (circRNAs) form circular structures via covalent bonds (71). CircRNAs play a crucial role in tumor progression, metastasis, and other malignant phenotypes. For example, circNHSL1 promotes gastric cancer progression through the miR-1306-3p/SIX1/vimentin axis (72). CircRNAs regulate iron and glutathione metabolism, lipid peroxidation, and mitochondria-associated proteins, all of which play key roles in iron-related death. Jiang et al. found that CircLRFN5 inhibited the growth of GBM through PRRX2/GCH1-mediated ferroptosis (73).

#### 2.4.5 GPX4

As an enzyme member of the GPX family, GPX4 protects cells from oxidative damage caused by ROS, thereby maintaining cellular lipid homeostasis. GPX4 not only acts as an important regulator of ferroptosis but also converts lipid hydroperoxides to lipid alcohols and prevents these molecules from triggering lipid peroxidation (74). As a potential therapeutic target for glioma, recent studies have shown that RAS-selective lethal 3 drives iron death through NF-κB pathway activation and GPX4 depletion in GBM (63).

Ferroptosis, a recently popular topic, has made great progress in the treatment of glioma, and its regulators Erastin, RSL3, RAS, and SLC7A11 have been studied in glioma. In conclusion, our experimental results provide relevant targets and treatment plans for future glioma treatment.

### 2.5 Pyroptosis

Pyroptosis, also known as cellular inflammatory necrosis, is a form of RCD characterized by continuous cellular distention until the cell membrane ruptures. This leads to the release of cellular contents, activating a strong inflammatory response (75). Cystathione activation is linked to necrosis and apoptosis. Initially, scorch death was thought to be related to cysteine-1 related cell death (76). Recent studies have shown that other cystathionases, such as cystathione-2/3/4/5/6/7/8/9/10/11, also induce damage in various cells and play significant roles in innate immunity and tumorigenesis.

Researchers conducted a comprehensive analysis of pyroptosis in glioma, utilizing the CGGA and TCGA databases, GDSC database, and GSVAR software. They concluded that pyroptosis-related genes effectively classified glioma patients well into two-dimensional distribution and that prognostic features based on these genes hold high clinical value. Finally, the expression of neuro CASP8 expression was higher in glioma compared to controls. The highest expression was observed in WHO IV, followed by WHO III, and the lowest in WHO II. Elevated CASP8 expression was associated with poor overall survival. These results suggest that CASP8 plays an oncogenic role in gliomas (77). Pyroptosis levels strongly indicate that the thermal tumor immune microenvironment has a high presence of CD8 ^+^ T cells and other T cell subtypes, as well as activation of P53 pathway, DNA repair, KRAS signaling, epithelial-mesenchymal transformation (EMT), IL6 JAK STAT3 signaling, IL2 STAT5 signaling, PI3K signaling, AKT signaling, mTOR signaling, and oncogenic pathways are enriched in the pyroptosis-Hi subgroup of cancer (132).

However, the role of pyroptosis in glioma has been less studied, and a comprehensive analysis of pyroptosis regulators in glioma, their correlation with clinical features, and their prognostic value has not yet been reported.

### 2.6 Necrosis

Necrosis is the death of local tissue cells *in vivo* and is characterized by changes in enzymatic solubility. Its morphological features include cell expansion, cell membrane rupture, release of cell contents, gradual nuclear changes, and incomplete DNA degradation, all of which contribute to a severe local inflammatory response. Necroptosis can be induced by the TNF receptor superfamily (80), T-cell receptors (81), interferon receptors, Toll-like receptors (TLR) (82), cellular metabolism, genotoxic stress, or the activation of various anticancer drugs, such as necrosis inhibitor-1 (Nec-1) (83).

#### 2.6.1 TNF receptor superfamily

Tumor necrosis factor (TNF/TNFα) is a type II transmembrane protein, with its intracellular amino terminus located at position (84). There are two types of TNF receptors: TNFR1 and TNFR2. TNFR1, which is present in most cells of the body, is activated by soluble ligands. TNFR2, which is mainly expressed in hematopoietic cells, primarily binds to the transmembrane TNF. The malignant cell-autonomous network of inflammatory cytokines includes TNF, the chemokine stromal cell-derived factor (SDF1, also known as CXCL12) and CCL2 (C-C chemokine ligand 2), cytokines IL-6 and macrophage inhibitory factor (MIF), and vascular endothelial growth factor (VEGF) (85). The TNF family has been shown to play a role in various types of tumors, including lung cancer (86) and ovarian cancer (87), and Tengfeng Yan et al. have demonstrated that TGF-β activates the TNF-α/NF-κB signaling pathway by inducing GBM mesenchymal transition through the upregulation of CLDN4 and nuclear translocation (88).

#### 2.6.2 TLR

The Toll-like receptor (TLR) family activates inflammatory response pathways that are crucial for effective immune cell recruitment. In the context of cancer, TLRs have tumor- and cell-type-specific pro- and antitumorigenic effects (89). The antitumorigenic effect of TLR is typically attributed to the stimulation of antitumor immunity through the activation of dendritic cells. This effect has been demonstrated to play a role in various cancers, including colorectal cancer (96) and bladder cancer (90). Alvarado et al. demonstrated that GBM cancer stem cells gain a survival advantage in challenging environments by diminishing their capacity to detect damage signals and activate innate immune responses through TLR4 (91).

Recent studies have shown that many regulatory factors such as the TNF family, TLR, and other drugs may induce necrosis, and the role of necrosis in lung cancer (86), ovarian cancer (87), colorectal cancer (96), bladder cancer (90), and glioma (91) has been shown to be evident in various research experiments.

### 2.7 Parthanayos

Parthanayos is a PARP1-dependent, cysteine-dependent cell death pathway characterized by chromosome condensation and DNA fragmentation. Many molecules, including PARP1, PARG, ARH3, AIF, and MIF (97).

PARP-1, also known as poly (ADP-ribose) synthase 1 or poly (ADP-ribose) transferase 1, exhibits higher basal levels in glioma cells compared to neurons and is positively associated with glioma malignancy and poor patient survival (98). Inhibiting PARP-1 has been a strategy for developing new drugs to overcome the resistance of glioma cells to radiotherapy or chemotherapy-induced apoptosis. Deoxypodophyllotoxin (DPT), the primary lignan and active component of the traditional plant camphor, suppresses the viability of glioma cells and induces their death by generating an excess of reactive oxygen species (ROS), which leads to abnormal germination of glioma cells (99).

Parthanayos is mainly dependent on PARP1-dependent and cysteine aspartic proteases, and recent studies have shown a clear role of PPAR1 in neurons. Inhibiting PARP-1 has been a strategy for developing new drugs to overcome the resistance of glioma cells to radiation- or chemotherapy-induced apoptosis. Current studies show that drugs like DPT (99) also play a role in treating GBM, offering the potential for glioma treatment.

### 2.8 Lysosome-dependent cell death

Lysosome-dependent cell death (LCD), also known as lysosomal cell death, is a form of RCD mediated by hydrolases (histones) or iron released by lysosome membrane permeabilization (LMP), characterized by lysosomal rupture (100), including Cathepsins, STAT3, TP53, NF-κB, MCOLN1 and other regulators (100). The results of Wei Zhou et al. showed that a newly synthesized lysosomal biological agent, Lys05, induced lysosomal membrane permeabilization in an LMP-dependent manner and increased radiosensitivity in GBM (101), thereby enhancing the therapeutic efficacy for glioma.

### 2.9 Cuproptosis

The main process of cuproptosis depends on the accumulation of intracellular Cu ions. Cu ions can directly bind to the lipid-acylated components of the tricarboxylic acid (TCA) cycle, leading to blockage. As a result, the accumulation and dysregulation of these proteins can lead to proteotoxic stress and ultimately cell death (105, 106). The primary regulators of coproptosis include FDX1, proteolipid acylation, DLAT, LIAS, pyruvate, α-ketoglutarate, and HSP70.

Cuproptosis has now been shown to have a role in cancers such as breast cancer (106). Wang et al. predicted that cuproptosis would likely help predict the prognosis, biological characteristics, and appropriate treatment of patients with glioma (107). Cuproptosis provides new targets and approaches for glioma treatment. Human H-ferritin (HFn), regorafenib, and Cu^2+^ were rationally designed as brain-targeted nanoplatforms (HFn-Cu-REGO NPs), with the aim of enhancing the effectiveness of Cu^2+^ and regorafenib in the treatment of GBM by modulating autophagy and prolapse (108).

Cuproptosis is an RCD modality that has only been proposed in the past two years and induces cell death through the TCA cycle in the mitochondria. Various researchers have predicted that cuproptosis may influence the prognosis and drug effectiveness in patients with glioma, offering a new target and approach for treating the condition.

## 3 Natural small-molecule compounds inhibit glioma through the modulation of different types of RCD

**Figure 4:**
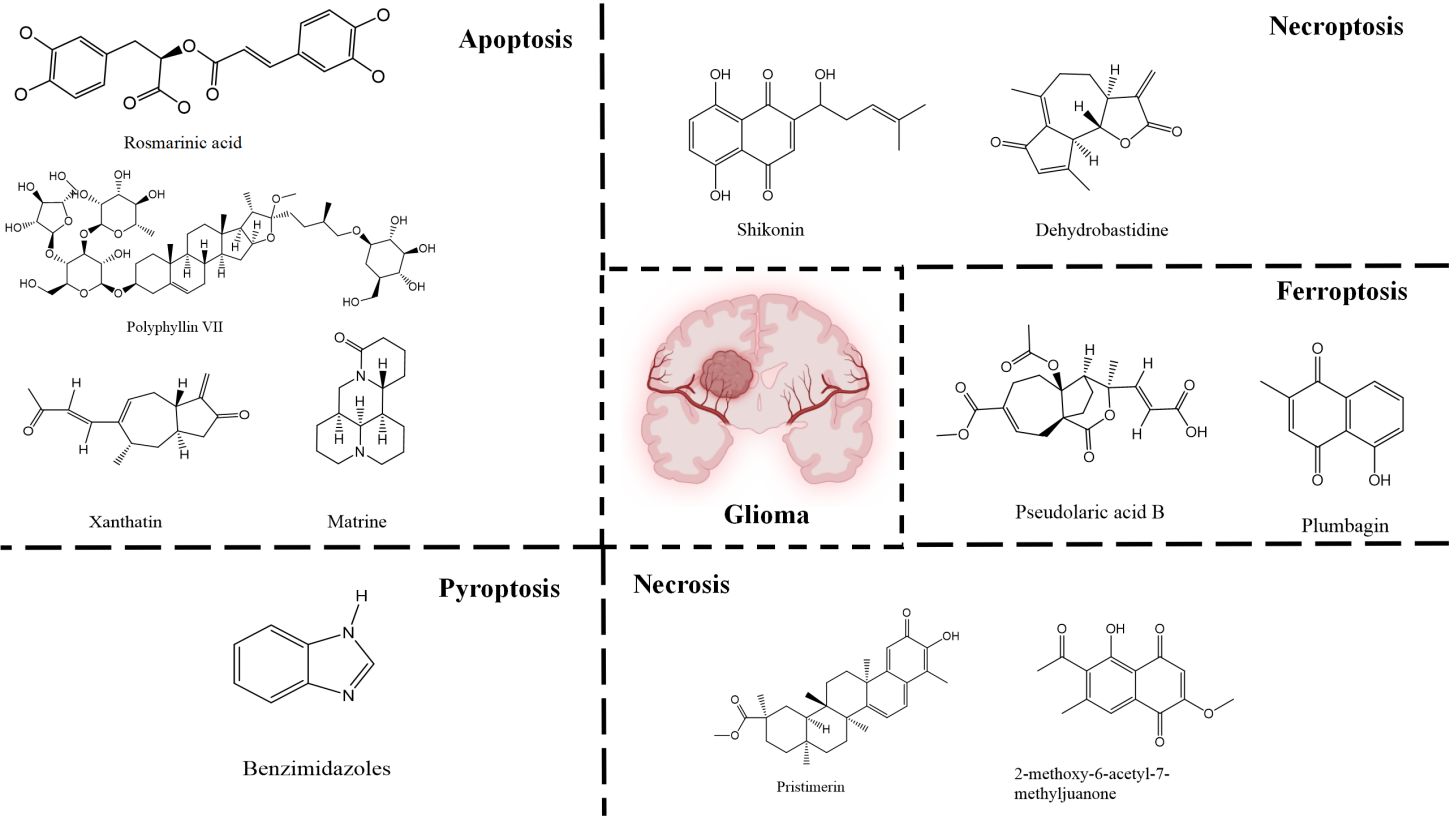
Natural small-molecule compounds for the treatment of glioma through different types of RCD.

The diversity of natural compounds provides a wide range of structures that can be used to develop libraries of compounds that can be used for future drug development. New technologies such as combinatorial synthesis and high-throughput screening have approved more than 50 percent of natural cancer drugs (133). Many natural drugs, such as quercetin (134) a role in gliomas. Next, we discuss how natural drugs (**Figure 4**, **Table 4**) inhibit glioma by inducing RCD.

**Table 4:** The anti-cancer natural small-molecule compounds in glioma by different types of RCD.

Natural small-molecule	Suppressive effect	Types of RCD	Function study	Stage	Cancer types	Reference
Rosmarinic acid 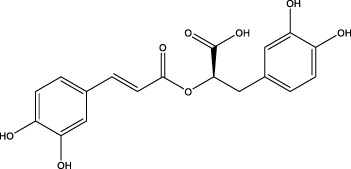	Inhibit proliferation, invasion, and induce apoptosis	Apoptosis	*In vitro*	Pre-clinical	GBM	(24)
Matrine 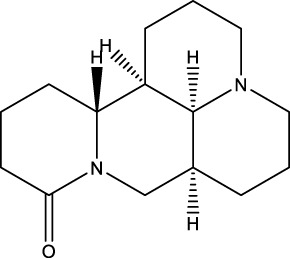	Induce apoptosis and autophagy	Apoptosis	*In vitro*	Pre-clinical	GBM	(25)
Xanthatin 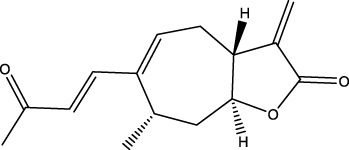	Induce apoptosis and inhibits tumor growth	Apoptosis	*In vitro and in vivo*	Pre-clinical	GBM	(26)
Polyphyllin VII 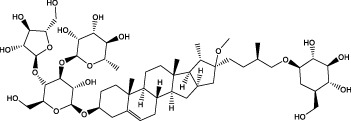	Induces cells death and autophagy	Apoptosis	*In vitro*	Pre-clinical	Astrooblastoma and GBM	(27)
Shikonin 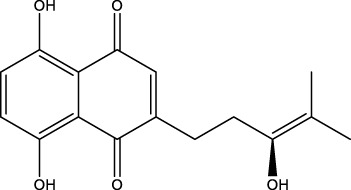	Induce apoptosis and necroptosis	Necroptosis	*In vitro*	Pre-clinical	Astrooblastoma	(35)
Dehydrobastidine 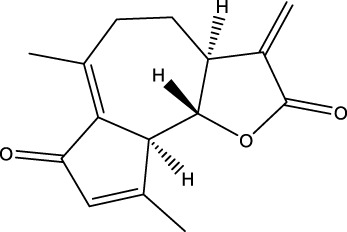	Induce apoptosis, autophagy, and necroptosis	Necroptosis	*In vitro*	Pre-clinical	Astrooblastoma and GBM	(36)
Pseudolaric acid B 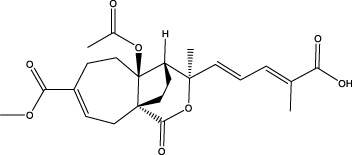	Inhibit the viabilities and induce ferroptosis	Ferroptosis	*In vitro and in vivo*	Pre-clinical	Astrooblastoma and GBM	(68)
Plumbagin 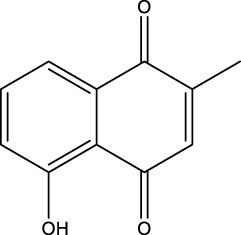	Inhibit the viabilities and induce ferroptosis	Ferroptosis	*In vitro and in vivo*	Pre-clinical	Astrooblastoma and GBM	(69)
Benzimidazoles 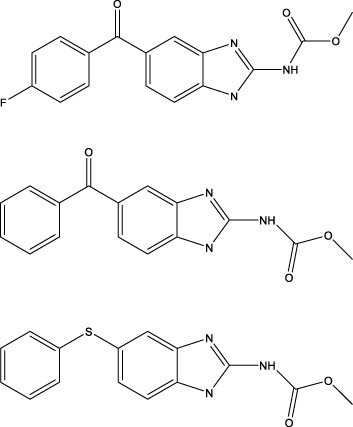	Induce apoptosis and pyroptosis and block the cell cycle	Pyroptosis	*In vitro*	Pre-clinical	GBM	(78)
Isobavitazarone 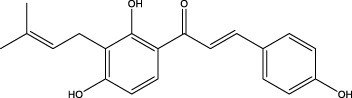	Inhibit proliferation, migration, and invasion and induce pyroptosis	Pyroptosis	*In vitro and in vivo*	Pre-clinical	GBM	(79)
Pristimerin 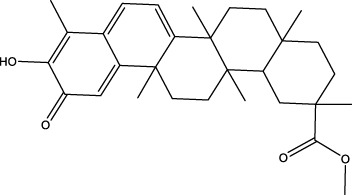	Inhibit the viabilities and growth and induce necrosis	Necrosis	*In vitro*	Pre-clinical	Astrooblastoma and GBM	(94)
2-methoxy-6-acetyl-7-methyljuanone 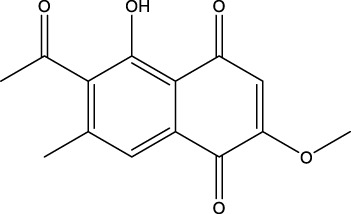	Inhibit the viabilities and growth and induce necrosis	Necrosis	*In vitro and in vivo*	Pre-clinical	GBM	(95)

### 3.1 Natural small-molecule compounds suppress glioma through apoptosis

Apoptosis is one of the most extensively researched forms of RCD. Several natural compounds have been found to induce apoptosis and inhibit the growth of glioma, including rosmarinic acid (RA) (24), matrine (25), xanthatin (26), and Polyphyllin VII (PP7) (27), etc. RA, a natural compound primarily found in the leaves of Rosmarinus officinalis, has demonstrated efficacy in inhibiting proliferation, invasion, and inducing apoptosis of GBM cells through the PI3K/Akt/NF−κB pathway (24). Matrine that has a variety of pharmacological properties, such as anti-inflammatory, antioxidant, and anti-fibrotic effects. It can also inhibit the growth of various cancer cells, including those of stomach cancer, chronic myelogenous leukemia, and breast cancer, by inducing apoptosis and autophagy. This is achieved through the downregulation of circ-104075 and Bcl-9 expression, which inhibits the PI3K/AKT and Wnt-β-catenin pathways in GBM cells (25). Xanthatin, a natural bioactive sesquiterpene lactone that has anti-angiogenic, antiviral, anti-inflammatory, antifungal, antibacterial effects and plays an anti-cancer role in non-small cell lung cancer, stomach cancer, breast cancer, melanoma and leukemia isolated from the aerial part of *Xanthium strumarium L.*, induces apoptosis and inhibits tumor growth via activating the endoplasmic reticulum stress-dependent CHOP pathway (26). PP7, a class of saponins that has anti-cancer effects in liver, lung, breast and colorectal cancer cells isolated from *Paris polyphylla* var. *yunnanensis* induces astrooblastoma and GBM cells death and autophagy via AKT/mTORC1 signaling (27). These natural drugs can inhibit the growth of gliomas by inducing apoptosis, and thus become backup drugs for the treatment of gliomas.

### 3.2 Natural small-molecule compounds restrain glioma growth via necroptosis

Furthermore, multiple studies have demonstrated that drugs can trigger necroptosis and effectively suppress the growth of gliomas. For example, Shikonin, a natural naphthoquinone isolated from the roots of *Lithospermum erythrorhizon*, can induce apoptosis and necrotic of GBM cells (35) by inhibiting EGFR phosphorylation and decreasing phosphorylation of EGFR downstream molecules, including AKT, P44/42MAPK and PLCγ1. Dehydrobastidine, a natural tumor seserpene lactone from *Artemisia douglassiana Besser* (Argentina) and *Gynoxys verrucosa* (Ecuador), has been shown to induce the phosphorylation of the tumor protein TP73. This process regulates apoptosis, autophagy, and necroptosis in astroblastoma and GBM cells (36), providing prospects for the treatment of glioma.

### 3.3 Natural small-molecule compounds inhibit glioma through ferroptosis

Furthermore, numerous natural products effectively inhibit the growth of gliomas and promote ferroptosis. For example, pseudolaric acid B (PAB), a diterpenoid acid that has anti-cancer effects in prostate cancer, cervical cancer and breast cancer isolated from the roots and bark of the trunk of the cortex of *Pseudolaric* pine, has been shown to trigger ferroptosis in astroblastoma and GBM cells by activating Nox4 and inhibiting xCT (68). Plumbagin (PLB; 5-hydroxy-2-methyl-1,4-naphthoquinone) inhibits GBM growth *in vitro and in vivo* through ferroptosis mediated by targeting NAD(P)H quinone dehydrogenase 1 (NQO1) and GPX4 (69).

### 3.4 Natural small-molecule compounds show promise in inducing pyroptosis in glioma cells

Understanding the mechanisms that underlie pyroptosis in cancer will contribute to developing new therapeutic strategies and clinical transformations. For drug effects, the compound benzimidazoles can induce apoptosis and pyroptosis of human GBM cells by blocking the cell cycle (78), while the natural drug isobavitazarone (IBC). as a compound isolated from *Psoralea corylifolia Linn* seeds, can relieve pyroptosis and thus contribute to enhancing apoptosis of GBM cells (79). Moreover, IBC exerts an anti-cancer effect on leukemia, colorectal cancer, liver cancer, breast cancer, prostate cancer, stomach cancer, cervical cancer, ovarian cancer, tongue squamous cell carcinoma and myeloma.

### 3.5 Natural small-molecule compounds display a suppressive effect on glioma through necrosis

In the field of drug research, numerous studies have demonstrated that natural compounds can trigger necrosis and inhibit the growth of cancer. For example, berberine was isolated from Aim. For example, berberine was isolated from *Aim. Rosin* can induce necrosis in prostate cancer cells (92) and Icaritin can trigger the necrosis of colorectal cancer cells (93). In gliomas, the natural product pristimerin, a natural quinonemethide triterpenoid isolated from various plant species in the *Celastraceae* and *Hippocrateaceae* families, activates c-Jun terminal kinase to induce apoptosis in astroblastoma and GBM cells, leading to programmed necrosis (94). Pristimerin also has anti-cancer effects in ovarian cancer, hepatocellular carcinoma, cervical cancer and breast cancer. 2-methoxy-6-acetyl-7-methyljuanone (MAM) that induced necrotic apoptosis of lung cancer and colorectal cancer cells inhibited glioma growth by inducing programmed necrosis of GBM cells by targeting NQO1 (95). This finding also offers potential for future glioma treatment.

Some naturally sourced compounds with specific molecular targets have shown promising therapeutic effects by blocking signaling proteins that promote cancer development. We summarized the relationship between natural drugs and RCD (such as apoptosis, necroptosis, pyroptosis, necrosis and ferroptosis) to achieve better precise treatment and clinical effects. These RCD related targeting drugs have been proven in the treatment of GBM, and the effect of other regulatory methods on glioma remains to be proven. Therefore, natural small molecule compounds may become clinical agents of GBM by regulating RCD.

## 4 Conclusion and perspective

Glioma, a persistent form of cancer, still has a low survival rate and poor prognosis. RCD is the primary mechanism through which organisms eliminate damaged cells that are at risk of tumor transformation or cells hijacked by microorganisms for pathogen replication. This process involves apoptosis apoptosis, and necroptosis, which act as natural barriers that limit the survival and propagation of malignant cells. Previous studies have shown that gliomas can be treated with RCD. RCD includes apoptosis, necroptosis, autophagy, ferroptosis, pyroptosis, necrosis, parathyroid hormone, entosis, lysosome-dependent cell death, NETosis, oxiptosis, alkaliptosis, cuproptosis, and disulfideptosis. In addition to the different forms of RCD that inhibit glioma growth described in this review, other forms of RCD have been found to have in gliomas, such as entosis (109, 110), NETosis (102, 103), oxeiptosis (111), alkaliptosis (112, 113) and disulfidptosis (114). Particularly, disulfidoptosis, a newly discovered RCD mode in 2022, may target the weakness of cancer metabolism (135). Although these forms of RCD have not been shown to be effective against gliomas, they have been shown to be effective against diseases such as breast cancer (136, 137), endometrial cancer (104), and pancreatic cancer (112, 113). Given their role in modulating cancer development and oncogenes in various types of cancer, the therapeutic potential of these compounds extends to other tumors, particularly gliomas, making them promising subjects for further research and potential treatment.

None of the treatments for glioma have been able to provide a complete cure. In the case of glioma treatment candidates, recent studies have found that, in addition to the commonly used drug temozolomide, numerous natural drugs such as RA (24), matrine (25), xanthatin (26, 47), PP7 (27), Shikonin (36), PAB (68), PLB (69), IBC (79), Pristimerin (94), MAM (95), DPT (99), etc. inhibit the growth of interlacing and inhibiting glioma by inducing apoptosis, necroptosis, autophagy, ferroptosis, pyroptosis, necrosis and parthanayos. The therapeutic potential of these natural small-molecule compounds in treating glioma makes them promising candidates for targeted therapeutic intervention. Future research may lead to a cure for gliomas. Although many drugs targeting RCD have not yet been approved, future therapeutic effects in gliomas can be anticipated based on previous analyses of the PubMed and Web of Science databases, as well as the effects observed in other tumors. In this review, we focus on the research progress of RCD in glioma. The aim is to develop new strategies and approaches for the treatment of glioma with the ultimate goal of achieving a cure in the future.

## Author contributions

MH: Writing – original draft. SL: Writing – review & editing. HF: Writing – review & editing. JA: Writing – review & editing. FP: Project administration, Supervision, Funding acquisition, Writing – review & editing. CP: Funding acquisition, Writing – review & editing.

## References

[1] Louis DN Perry A Wesseling P Brat DJ Cree IA Figarella-Branger D. The 2021 WHO classification of tumors of the central nervous system: a summary. Neuro-Oncol (2021) 23:1231–51. doi: 10.1093/neuonc/noab106 PMC8328013 34185076

[2] Weller M Le Rhun E. How did lomustine become standard of care in recurrent glioblastoma? Cancer Treat Rev (2020) 87:102029. doi: 10.1016/j.ctrv.2020.102029 32408220

[3] Wrensch M Minn Y Chew T Bondy M Berger MS. Epidemiology of primary brain tumors: current concepts and review of the literature. Neuro-Oncol (2002) 4:278–99. doi: 10.1093/neuonc/4.4.278 PMC1920665 12356358

[4] Nakada M Kita D Watanabe T Hayashi Y Teng L Pyko IV. Aberrant signaling pathways in glioma. Cancers (2011) 3:3242–78. doi: 10.3390/cancers3033242 PMC3759196 24212955

[5] Vartanian A Singh SK Agnihotri S Jalali S Burrell K Aldape KD. GBM’s multifaceted landscape: highlighting regional and microenvironmental heterogeneity. Neuro-Oncol (2014) 16:1167–75. doi: 10.1093/neuonc/nou035 PMC4136895 24642524

[6] Stupp R Mason WP van den Bent MJ Weller M Fisher B Taphoorn MJB. Radiotherapy plus concomitant and adjuvant temozolomide for glioblastoma. N Engl J Med (2005) 352:987–96. doi: 10.1056/NEJMoa043330 15758009

[7] Kaufmann SH Gores GJ. Apoptosis in cancer: cause and cure. BioEssays (2000) 22:1007–17. doi: 10.1002/1521-1878(200011)22:11<1007::AID-BIES7>3.0.CO;2-4 11056477

[8] Bedoui S Herold MJ Strasser A. Emerging connectivity of programmed cell death pathways and its physiological implications. Nat Rev Mol Cell Biol (2020) 21:678–95. doi: 10.1038/s41580-020-0270-8 32873928

[9] Su Z Yang Z Xu Y Chen Y Yu Q. Apoptosis, autophagy, necroptosis, and cancer metastasis. Mol Cancer (2015) 14:48. doi: 10.1186/s12943-015-0321-5 25743109 PMC4343053

[10] Kastenhuber E Lowe S. Putting p53 in context. Cell (2017) 170:1062–78. doi: 10.1016/j.cell.2017.08.028 PMC5743327 28886379

[11] Lou J Hao Y Lin K Lyu Y Chen M Wang H. Circular RNA CDR1as disrupts the p53/MDM2 complex to inhibit Gliomagenesis. Mol Cancer (2020) 19:138. doi: 10.1186/s12943-020-01253-y 32894144 PMC7487905

[12] Safa AR. c-FLIP, a master anti-apoptotic regulator. Exp Oncol (2012) 34:176–84. PMC4817998 23070002

[13] Wu Y-J Wu Y-H Mo S-T Hsiao H-W He Y-W Lai M-Z. Cellular FLIP inhibits myeloid cell activation by suppressing selective innate signaling. J Immunol Baltim Md 1950 (2015) 195:2612–23. doi: 10.4049/jimmunol.1402944 26238491

[14] Ishioka T Katayama R Kikuchi R Nishimoto M Takada S Takada R. Impairment of the ubiquitin-proteasome system by cellular FLIP. Genes Cells Devoted Mol Cell Mech (2007) 12:735–44. doi: 10.1111/j.1365-2443.2007.01087.x 17573774

[15] Kreuz S Siegmund D Rumpf J-J Samel D Leverkus M Janssen O. NFkappaB activation by Fas is mediated through FADD, caspase-8, and RIP and is inhibited by FLIP. J Cell Biol (2004) 166:369–80. doi: 10.1083/jcb.200401036 PMC2172264 15289496

[16] Nakajima A Komazawa-Sakon S Takekawa M Sasazuki T Yeh W-C Yagita H. An antiapoptotic protein, c-FLIPL, directly binds to MKK7 and inhibits the JNK pathway. EMBO J (2006) 25:5549–59. doi: 10.1038/sj.emboj.7601423 PMC1679768 17110930

[17] Zhu Z-C Liu J-W Yang C Li M-J Wu R-J Xiong Z-Q. Targeting KPNB1 overcomes TRAIL resistance by regulating DR5, Mcl-1 and FLIP in glioblastoma cells. Cell Death Dis (2019) 10:118. doi: 10.1038/s41419-019-1383-x 30742128 PMC6370806

[18] Fulda S Vucic D. Targeting IAP proteins for therapeutic intervention in cancer. Nat Rev Drug Discovery (2012) 11:109–24. doi: 10.1038/nrd3627 22293567

[19] Zappavigna S Scuotto M Cossu AM Ingrosso D De Rosa M Schiraldi C. The 1,4 benzoquinone-featured 5-lipoxygenase inhibitor RF-Id induces apoptotic death through downregulation of IAPs in human glioblastoma cells. J Exp Clin Cancer Res CR (2016) 35:167. doi: 10.1186/s13046-016-0440-x 27770821 PMC5075202

[20] Yip KW Reed JC. Bcl-2 family proteins and cancer. Oncogene (2008) 27:6398–406. doi: 10.1038/onc.2008.307 18955968

[21] Carneiro BA El-Deiry WS. Targeting apoptosis in cancer therapy. Nat Rev Clin Oncol (2020) 17:395–417. doi: 10.1038/s41571-020-0341-y 32203277 PMC8211386

[22] Liang J Cao R Wang X Zhang Y Wang P Gao H. Mitochondrial PKM2 regulates oxidative stress-induced apoptosis by stabilizing Bcl2. Cell Res (2017) 27:329–51. doi: 10.1038/cr.2016.159 PMC5339831 28035139

[23] Levesley J Steele L Brüning-Richardson A Davison A Zhou J Ding C. Selective BCL-XL inhibition promotes apoptosis in combination with MLN8237 in medulloblastoma and pediatric glioblastoma cells. Neuro-Oncol (2018) 20:203–14. doi: 10.1093/neuonc/nox134 PMC7059858 29016820

[24] Liu Y Xu X Tang H Pan Y Hu B Huang G. Rosmarinic acid inhibits cell proliferation, migration, and invasion and induces apoptosis in human glioma cells. Int J Mol Med (2021) 47:67. doi: 10.3892/ijmm.2021.4900 33649774 PMC7952246

[25] Chi G Xu D Zhang B Yang F. Matrine induces apoptosis and autophagy of glioma cell line U251 by regulation of circRNA-104075/BCL-9. Chem Biol Interact (2019) 308:198–205. doi: 10.1016/j.cbi.2019.05.030 31112718

[26] Ma Y-Y Di Z-M Cao Q Xu W-S Bi S-X Yu J-S. Xanthatin induces glioma cell apoptosis and inhibits tumor growth *via* activating endoplasmic reticulum stress-dependent CHOP pathway. Acta Pharmacol Sin (2020) 41:404–14. doi: 10.1038/s41401-019-0318-5 PMC7468336 31700088

[27] Pang D Li C Yang C Zou Y Feng B Li L. Polyphyllin VII promotes apoptosis and autophagic cell death *via* ROS-inhibited AKT activity, and sensitizes glioma cells to temozolomide. Oxid Med Cell Longev (2019) 2019:1805635. doi: 10.1155/2019/1805635 31814867 PMC6877958

[28] Thayyullathil F Cheratta AR Pallichankandy S Subburayan K Tariq S Rangnekar VM. Par-4 regulates autophagic cell death in human cancer cells *via* upregulating p53 and BNIP3. Biochim Biophys Acta Mol Cell Res (2020) 1867:118692. doi: 10.1016/j.bbamcr.2020.118692 32135176

[29] Galluzzi L Vitale I Abrams JM Alnemri ES Baehrecke EH Blagosklonny MV. Molecular definitions of cell death subroutines: recommendations of the Nomenclature Committee on Cell Death 2012. Cell Death Differ (2012) 19:107–20. doi: 10.1038/cdd.2011.96 PMC3252826 21760595

[30] Feng X Song Q Yu A Tang H Peng Z Wang X. Receptor-interacting protein kinase 3 is a predictor of survival and plays a tumor suppressive role in colorectal cancer. Neoplasma (2015) 62:592–601. doi: 10.4149/neo_2015_071 25997957

[31] Quinn JJ Chang HY. Unique features of long non-coding RNA biogenesis and function. Nat Rev Genet (2016) 17:47–62. doi: 10.1038/nrg.2015.10 26666209

[32] Jiang F Zhan Z Yang Y Liu G Liu S Gu J. Construction and validation of a necroptosis-related lncRNA signature in prognosis and immune microenvironment for glioma. J Oncol (2022) 2022:e5681206. doi: 10.1155/2022/5681206 PMC9440826 36065303

[33] Kanduc D Mittelman A Serpico R Sinigaglia E Sinha AA Natale C. Cell death: apoptosis versus necrosis (review). Int J Oncol (2002) 21:165–70. doi: 10.3892/ijo.21.1.165 12063564

[34] Ding Y He C Lu S Wang X Wang C Wang L. MLKL contributes to shikonin-induced glioma cell necroptosis *via* promotion of chromatinolysis. Cancer Lett (2019) 467:58–71. doi: 10.1016/j.canlet.2019.09.007 31560934

[35] Zhao Q Kretschmer N Bauer R Efferth T. Shikonin and its derivatives inhibit the epidermal growth factor receptor signaling and synergistically kill glioblastoma cells in combination with erlotinib. Int J Cancer (2015) 137:1446–56. doi: 10.1002/ijc.29483 25688715

[36] Ratovitski EA. Dehydroleucodine induces a TP73-dependent transcriptional regulation of multiple cell death target genes in human glioblastoma cells. Anticancer Agents Med Chem (2017) 17:839–50. doi: 10.2174/1871520616666160923105546 27671304

[37] Zhou Z Xu J Huang N Tang J Ma P Cheng Y. Clinical and biological significance of a necroptosis-related gene signature in glioma. Front Oncol (2022) 12:855434. doi: 10.3389/fonc.2022.855434 35719998 PMC9201102

[38] Ohsumi Y. Historical landmarks of autophagy research. Cell Res (2014) 24:9–23. doi: 10.1038/cr.2013.169 24366340 PMC3879711

[39] Levy JMM Towers CG Thorburn A. Targeting autophagy in cancer. Nat Rev Cancer (2017) 17:528–42. doi: 10.1038/nrc.2017.53 PMC5975367 28751651

[40] Yang ZJ Chee CE Huang S Sinicrope FA. The role of autophagy in cancer: therapeutic implications. Mol Cancer Ther (2011) 10:1533–41. doi: 10.1158/1535-7163.MCT-11-0047 PMC3170456 21878654

[41] Li J Yen C Liaw D Podsypanina K Bose S Wang SI. PTEN, a putative protein tyrosine phosphatase gene mutated in human brain, breast, and prostate cancer. Science (1997) 275:1943–7. doi: 10.1126/science.275.5308.1943 9072974

[42] Petiot A Ogier-Denis E Blommaart EF Meijer AJ Codogno P. Distinct classes of phosphatidylinositol 3’-kinases are involved in signaling pathways that control macroautophagy in HT-29 cells. J Biol Chem (2000) 275:992–8. doi: 10.1074/jbc.275.2.992 10625637

[43] Benitez JA Finlay D Castanza A Parisian AD Ma J Longobardi C. PTEN deficiency leads to proteasome addiction: a novel vulnerability in glioblastoma. Neuro-Oncol (2021) 23:1072–86. doi: 10.1093/neuonc/noab001 PMC8661409 33428749

[44] Lemmon MA Schlessinger J Ferguson KM. The EGFR family: not so prototypical receptor tyrosine kinases. Cold Spring Harb Perspect Biol (2014) 6:a020768. doi: 10.1101/cshperspect.a020768 24691965 PMC3970421

[45] Palumbo S Tini P Toscano M Allavena G Angeletti F Manai F. Combined EGFR and autophagy modulation impairs cell migration and enhances radiosensitivity in human glioblastoma cells. J Cell Physiol (2014) 229:1863–73. doi: 10.1002/jcp.24640 24691646

[46] Laplante M Sabatini DM. mTOR signaling in growth control and disease. Cell (2012) 149:274–93. doi: 10.1016/j.cell.2012.03.017 PMC3331679 22500797

[47] Chen H Zhu T Huang X Xu W Di Z Ma Y. Xanthatin suppresses proliferation and tumorigenicity of glioma cells through autophagy inhibition *via* activation of the PI3K-Akt-mTOR pathway. Pharmacol Res Perspect (2023) 11:e01041. doi: 10.1002/prp2.1041 36572650 PMC9792428

[48] Jiang Y Liu J Hong W Fei X Liu R. Arctigenin inhibits glioblastoma proliferation through the AKT/mTOR pathway and induces autophagy. BioMed Res Int (2020) 2020:3542613. doi: 10.1155/2020/3542613 33015162 PMC7512051

[49] Liu X Zhao P Wang X Wang L Zhu Y Song Y. Celastrol mediates autophagy and apoptosis via the ROS/JNK and Akt/mTOR signaling pathways in glioma cells. J Exp Clin Cancer Res CR (2019) 38:184. doi: 10.1186/s13046-019-1173-4 31053160 PMC6500040

[50] Zheng X Li W Xu H Liu J Ren L Yang Y. Sinomenine ester derivative inhibits glioblastoma by inducing mitochondria-dependent apoptosis and autophagy by PI3K/AKT/mTOR and AMPK/mTOR pathway. Acta Pharm Sin B (2021) 11:3465–80. doi: 10.1016/j.apsb.2021.05.027 PMC8642618 34900530

[51] Li G Zhong Y Wang W Jia X Zhu H Jiang W. Sempervirine mediates autophagy and apoptosis *via* the Akt/mTOR signaling pathways in glioma cells. Front Pharmacol (2021) 12:770667. doi: 10.3389/fphar.2021.770667 34916946 PMC8670093

[52] Wang J Qi Q Zhou W Feng Z Huang B Chen A. Inhibition of glioma growth by flavokawain B is mediated through endoplasmic reticulum stress induced autophagy. Autophagy (2018) 14:2007–22. doi: 10.1080/15548627.2018.1501133 PMC6152528 30025493

[53] Chen Y Li N Wang H Wang N Peng H Wang J. Amentoflavone suppresses cell proliferation and induces cell death through triggering autophagy-dependent ferroptosis in human glioma. Life Sci (2020) 247:117425. doi: 10.1016/j.lfs.2020.117425 32057904

[54] Qiao H Wang Y-B Gao Y-M Bi L-L. Prucalopride inhibits the glioma cells proliferation and induces autophagy *via* AKT-mTOR pathway. BMC Neurol (2018) 18:80. doi: 10.1186/s12883-018-1083-7 29866060 PMC5985575

[55] Liu X Zhao P Wang X Wang L Zhu Y Gao W. Triptolide induces glioma cell autophagy and apoptosis *via* upregulating the ROS/JNK and downregulating the Akt/mTOR signaling pathways. Front Oncol (2019) 9:387. doi: 10.3389/fonc.2019.00387 31157167 PMC6528693

[56] Wang N Zhang Q Luo L Ning B Fang Y. β-asarone inhibited cell growth and promoted autophagy *via* P53/Bcl-2/Bclin-1 and P53/AMPK/mTOR pathways in Human Glioma U251 cells. J Cell Physiol (2018) 233:2434–43. doi: 10.1002/jcp.26118 28776671

[57] Jiang X Stockwell BR Conrad M. Ferroptosis: mechanisms, biology and role in disease. Nat Rev Mol Cell Biol (2021) 22:266–82. doi: 10.1038/s41580-020-00324-8 PMC8142022 33495651

[58] Yagoda N von Rechenberg M Zaganjor E Bauer AJ Yang WS Fridman DJ. RAS–RAF–MEK-dependent oxidative cell death involving voltage-dependent anion channels. Nature (2007) 447:865–9. doi: 10.1038/nature05859 PMC3047570 17568748

[59] Chen S Zhang Z Zhang B Huang Q Liu Y Qiu Y. CircCDK14 promotes tumor progression and resists ferroptosis in glioma by regulating PDGFRA. Int J Biol Sci (2022) 18:841–57. doi: 10.7150/ijbs.66114 PMC8741855 35002529

[60] Moore AR Rosenberg SC McCormick F Malek S. RAS-targeted therapies: is the undruggable drugged? Nat Rev Drug Discovery (2020) 19:533–52. doi: 10.1038/s41573-020-0068-6 PMC7809886 32528145

[61] Chen D Fan Z Rauh M Buchfelder M Eyupoglu IY Savaskan N. ATF4 promotes angiogenesis and neuronal cell death and confers ferroptosis in a xCT-dependent manner. Oncogene (2017) 36:5593–608. doi: 10.1038/onc.2017.146 PMC5633655 28553953

[62] Wang X Lu S He C Wang C Wang L Piao M. RSL3 induced autophagic death in glioma cells *via* causing glycolysis dysfunction. Biochem Biophys Res Commun (2019) 518:590–7. doi: 10.1016/j.bbrc.2019.08.096 31445705

[63] Li S He Y Chen K Sun J Zhang L He Y. RSL3 drives ferroptosis through NF-κB pathway activation and GPX4 depletion in glioblastoma. Oxid Med Cell Longev (2021) 2021:e2915019. doi: 10.1155/2021/2915019 PMC8720588 34987700

[64] Mao C Liu X Zhang Y Lei G Yan Y Lee H. DHODH-mediated ferroptosis defence is a targetable vulnerability in cancer. Nature (2021) 593:586–90. doi: 10.1038/s41586-021-03539-7 PMC8895686 33981038

[65] Sontheimer H Bridges RJ. Sulfasalazine for brain cancer fits. Expert Opin Investig Drugs (2012) 21:575–8. doi: 10.1517/13543784.2012.670634 PMC3644176 22404218

[66] Sun S Guo C Gao T Ma D Su X Pang Q. Hypoxia enhances glioma resistance to sulfasalazine-induced ferroptosis by upregulating SLC7A11 *via* PI3K/AKT/HIF-1α Axis. Oxid Med Cell Longev (2022) 2022:7862430. doi: 10.1155/2022/7862430 36439690 PMC9699746

[67] Zhao X Zhou M Yang Y Luo M. The ubiquitin hydrolase OTUB1 promotes glioma cell stemness via suppressing ferroptosis through stabilizing SLC7A11 protein. Bioengineered (2021) 12:12636–45. doi: 10.1080/21655979.2021.2011633 PMC8810032 34927544

[68] Wang Z Ding Y Wang X Lu S Wang C He C. Pseudolaric acid B triggers ferroptosis in glioma cells *via* activation of Nox4 and inhibition of xCT. Cancer Lett (2018) 428:21–33. doi: 10.1016/j.canlet.2018.04.021 29702192

[69] Zhan S Lu L Pan S Wei X Miao R Liu X. Targeting NQO1/GPX4-mediated ferroptosis by plumbagin suppresses in *vitro* and in *vivo* glioma growth. Br J Cancer (2022) 127:364–76. doi: 10.1038/s41416-022-01800-y PMC9296534 35396498

[70] Liu T Zhu C Chen X Guan G Zou C Shen S. Ferroptosis, as the most enriched programmed cell death process in glioma, induces immunosuppression and immunotherapy resistance. Neuro-Oncol (2022) 24:1113–25. doi: 10.1093/neuonc/noac033 PMC9248406 35148413

[71] Salami R Salami M Mafi A Vakili O Asemi Z. Circular RNAs and glioblastoma multiforme: focus on molecular mechanisms. Cell Commun Signal CCS (2022) 20:13. doi: 10.1186/s12964-021-00809-9 35090496 PMC8796413

[72] Zhu Z Rong Z Luo Z Yu Z Zhang J Qiu Z. Circular RNA circNHSL1 promotes gastric cancer progression through the miR-1306-3p/SIX1/vimentin axis. Mol Cancer (2019) 18:126. doi: 10.1186/s12943-019-1054-7 31438963 PMC6704702

[73] Jiang Y Zhao J Li R Liu Y Zhou L Wang C. CircLRFN5 inhibits the progression of glioblastoma *via* PRRX2/GCH1 mediated ferroptosis. J Exp Clin Cancer Res (2022) 41:307. doi: 10.1186/s13046-022-02518-8 36266731 PMC9583503

[74] Yang WS SriRamaratnam R Welsch ME Shimada K Skouta R Viswanathan VS. Regulation of ferroptotic cancer cell death by GPX4. Cell (2014) 156:317–31. doi: 10.1016/j.cell.2013.12.010 PMC4076414 24439385

[75] Yu P Zhang X Liu N Tang L Peng C Chen X. Pyroptosis: mechanisms and diseases. Signal Transduct Target Ther (2021) 6:1–21. doi: 10.1038/s41392-021-00507-5 33776057 PMC8005494

[76] Broz P Dixit VM. Inflammasomes: mechanism of assembly, regulation and signalling. Nat Rev Immunol (2016) 16:407–20. doi: 10.1038/nri.2016.58 27291964

[77] Chen P Li Y Li N Shen L Li Z. Comprehensive analysis of pyroptosis-associated in molecular classification, immunity and prognostic of glioma. Front Genet (2022) 12:781538. doi: 10.3389/fgene.2021.781538 35069683 PMC8777075

[78] Ren L-W Li W Zheng X-J Liu J-Y Yang Y-H Li S. Benzimidazoles induce concurrent apoptosis and pyroptosis of human glioblastoma cells *via* arresting cell cycle. Acta Pharmacol Sin (2022) 43:194–208. doi: 10.1038/s41401-021-00752-y 34433903 PMC8724275

[79] Wu Y Chang J Ge J Xu K Zhou Q Zhang X. Isobavachalcone’s alleviation of pyroptosis contributes to enhanced apoptosis in glioblastoma: possible involvement of NLRP3. Mol Neurobiol (2022) 59:6934–55. doi: 10.1007/s12035-022-03010-2 36053436

[80] Lu JV Chen HC Walsh CM. Necroptotic signaling in adaptive and innate immunity. Semin Cell Dev Biol (2014) 35:33–9. doi: 10.1016/j.semcdb.2014.07.003 PMC4197103 25042848

[81] Ch’en IL Tsau JS Molkentin JD Komatsu M Hedrick SM. Mechanisms of necroptosis in T cells. J Exp Med (2011) 208:633–41. doi: 10.1084/jem.20110251 PMC3135356 21402742

[82] He S Liang Y Shao F Wang X. Toll-like receptors activate programmed necrosis in macrophages through a receptor-interacting kinase-3-mediated pathway. Proc Natl Acad Sci U.S.A. (2011) 108:20054–9. doi: 10.1073/pnas.1116302108 PMC3250173 22123964

[83] Degterev A Huang Z Boyce M Li Y Jagtap P Mizushima N. Chemical inhibitor of nonapoptotic cell death with therapeutic potential for ischemic brain injury. Nat Chem Biol (2005) 1:112–9. doi: 10.1038/nchembio711 16408008

[84] Balkwill F. Tumour necrosis factor and cancer. Nat Rev Cancer (2009) 9:361–71. doi: 10.1038/nrc2628 19343034

[85] Suganuma M Okabe S Marino MW Sakai A Sueoka E Fujiki H. Essential role of tumor necrosis factor alpha (TNF-alpha) in tumor promotion as revealed by TNF-alpha-deficient mice. Cancer Res (1999) 59:4516–8. 10493498

[86] Stathopoulos GT Kollintza A Moschos C Psallidas I Sherrill TP Pitsinos EN. Tumor necrosis factor-alpha promotes Malignant pleural effusion. Cancer Res (2007) 67:9825–34. doi: 10.1158/0008-5472.CAN-07-1064 17942913

[87] Hagemann T Wilson J Burke F Kulbe H Li NF Plüddemann A. Ovarian cancer cells polarize macrophages toward a tumor-associated phenotype. J Immunol Baltim Md 1950 (2006) 176:5023–32. doi: 10.4049/jimmunol.176.8.5023 16585599

[88] Yan T Tan Y Deng G Sun Z Liu B Wang Y. TGF-β induces GBM mesenchymal transition through upregulation of CLDN4 and nuclear translocation to activate TNF-α/NF-κB signal pathway. Cell Death Dis (2022) 13:1–11. doi: 10.1038/s41419-022-04788-8 PMC9008023 35418179

[89] Pradere J-P Dapito DH Schwabe RF. The Yin and Yang of Toll-like receptors in cancer. Oncogene (2014) 33:3485–95. doi: 10.1038/onc.2013.302 PMC4059777 23934186

[90] Cheah MT Chen JY Sahoo D Contreras-Trujillo H Volkmer AK Scheeren FA. CD14-expressing cancer cells establish the inflammatory and proliferative tumor microenvironment in bladder cancer. Proc Natl Acad Sci USA (2015) 112:4725–30. doi: 10.1073/pnas.1424795112 PMC4403197 25825750

[91] Alvarado AG Thiagarajan PS Mulkearns-Hubert EE Silver DJ Hale JS Alban TJ. Glioblastoma cancer stem cells evade innate immune suppression of self-renewal through reduced TLR4 expression. Cell Stem Cell (2017) 20:450–461.e4. doi: 10.1016/j.stem.2016.12.001 28089910 PMC5822422

[92] Zhang G Jiang C Wang Z Chen W Gu W Ding Y. Dehydroabietic acid derivative QC2 induces oncosis in hepatocellular carcinoma cells. BioMed Res Int (2014) 2014:682197. doi: 10.1155/2014/682197 25110686 PMC4109319

[93] Zhou C Chen Z Lu X Wu H Yang Q Xu D. Icaritin activates JNK-dependent mPTP necrosis pathway in colorectal cancer cells. Tumour Biol J Int Soc Oncodevelopmental Biol Med (2016) 37:3135–44. doi: 10.1007/s13277-015-4134-3 26427664

[94] Zhao H Wang C Lu B Zhou Z Jin Y Wang Z. Pristimerin triggers AIF-dependent programmed necrosis in glioma cells *via* activation of JNK. Cancer Lett (2016) 374:136–48. doi: 10.1016/j.canlet.2016.01.055 26854718

[95] Yu J Zhong B Jin L Hou Y Ai N Ge W. 2-Methoxy-6-acetyl-7-methyljuglone (MAM) induced programmed necrosis in glioblastoma by targeting NAD(P)H: Quinone oxidoreductase 1 (NQO1). Free Radic Biol Med (2020) 152:336–47. doi: 10.1016/j.freeradbiomed.2020.03.026 32234332

[96] Grimm M Kim M Rosenwald A Heemann U Germer C-T Waaga-Gasser AM. Toll-like receptor (TLR) 7 and TLR8 expression on CD133+ cells in colorectal cancer points to a specific role for inflammation-induced TLRs in tumourigenesis and tumour progression. Eur J Cancer Oxf Engl 1990 (2010) 46:2849–57. doi: 10.1016/j.ejca.2010.07.017 20728343

[97] Zhou Y Liu L Tao S Yao Y Wang Y Wei Q. Parthanatos and its associated components: Promising therapeutic targets for cancer. Pharmacol Res (2021) 163:105299. doi: 10.1016/j.phrs.2020.105299 33171306

[98] Galia A Calogero AE Condorelli R Fraggetta F Corte AL Ridolfo F. PARP-1 protein expression in glioblastoma multiforme. Eur J Histochem (2012) 56:e9–9. doi: 10.4081/ejh.2012.e9 PMC3352138 22472897

[99] Ma D Lu B Feng C Wang C Wang Y Luo T. Deoxypodophyllotoxin triggers parthanatos in glioma cells *via* induction of excessive ROS. Cancer Lett (2016) 371:194–204. doi: 10.1016/j.canlet.2015.11.044 26683770

[100] Tang D Kang R Berghe TV Vandenabeele P Kroemer G. The molecular machinery of regulated cell death. Cell Res (2019) 29:347–64. doi: 10.1038/s41422-019-0164-5 PMC6796845 30948788

[101] Zhou W Guo Y Zhang X Jiang Z. Lys05 induces lysosomal membrane permeabilization and increases radiosensitivity in glioblastoma. J Cell Biochem (2020) 121:2027–37. doi: 10.1002/jcb.29437 31642111

[102] Yipp BG Kubes P. NETosis: how vital is it? Blood (2013) 122:2784–94. doi: 10.1182/blood-2013-04-457671 24009232

[103] Thiam HR Wong SL Wagner DD Waterman CM. Cellular mechanisms of NETosis. Annu Rev Cell Dev Biol (2020) 36:191–218. doi: 10.1146/annurev-cellbio-020520-111016 32663035 PMC8499668

[104] Ronchetti L Terrenato I Ferretti M Corrado G Goeman F Donzelli S. Circulating cell free DNA and citrullinated histone H3 as useful biomarkers of NETosis in endometrial cancer. J Exp Clin Cancer Res CR (2022) 41:151. doi: 10.1186/s13046-022-02359-5 35449078 PMC9027343

[105] Tang D Chen X Kroemer G. Cuproptosis: a copper-triggered modality of mitochondrial cell death. Cell Res (2022) 32:417–8. doi: 10.1038/s41422-022-00653-7 PMC9061796 35354936

[106] Tsvetkov P Coy S Petrova B Dreishpoon M Verma A Abdusamad M. Copper induces cell death by targeting lipoylated TCA cycle proteins. Science (2022) 375:1254–61. doi: 10.1126/science.abf0529 PMC9273333 35298263

[107] Wang W Lu Z Wang M Liu Z Wu B Yang C. The cuproptosis-related signature associated with the tumor environment and prognosis of patients with glioma. Front Immunol (2022) 13:998236. doi: 10.3389/fimmu.2022.998236 36110851 PMC9468372

[108] Jia W Tian H Jiang J Zhou L Li L Luo M. Brain-targeted HFn-Cu-REGO nanoplatform for site-specific delivery and manipulation of autophagy and cuproptosis in glioblastoma. Small Weinh Bergstr Ger (2023) 19:e2205354. doi: 10.1002/smll.202205354 36399643

[109] Kianfar M Balcerak A Chmielarczyk M Tarnowski L Grzybowska EA. Cell death by entosis: triggers, molecular mechanisms and clinical significance. Int J Mol Sci (2022) 23:4985. doi: 10.3390/ijms23094985 35563375 PMC9102690

[110] Overholtzer M Mailleux AA Mouneimne G Normand G Schnitt SJ King RW. A nonapoptotic cell death process, entosis, that occurs by cell-in-cell invasion. Cell (2007) 131:966–79. doi: 10.1016/j.cell.2007.10.040 18045538

[111] Holze C Michaudel C Mackowiak C Haas DA Benda C Hubel P. Oxeiptosis – a ROS induced caspase-independent apoptosis-like cell death pathway. Nat Immunol (2018) 19:130–40. doi: 10.1038/s41590-017-0013-y PMC5786482 29255269

[112] Song X Zhu S Xie Y Liu J Sun L Zeng D. JTC801 induces pH-dependent death specifically in cancer cells and slows growth of tumors in mice. Gastroenterology (2018) 154:1480–93. doi: 10.1053/j.gastro.2017.12.004 PMC5880694 29248440

[113] Liu J Kuang F Kang R Tang D. Alkaliptosis: a new weapon for cancer therapy. Cancer Gene Ther (2020) 27:267–9. doi: 10.1038/s41417-019-0134-6 31467365

[114] Liu X Nie L Zhang Y Yan Y Wang C Colic M. Actin cytoskeleton vulnerability to disulfide stress mediates disulfidptosis. Nat Cell Biol (2023) 25(3):404–14. doi: 10.1038/s41556-023-01091-2 PMC10027392 36747082

[115] Dzobo K. Epigenomics-guided drug development: recent advances in solving the cancer treatment “jigsaw puzzle ”. Omics J Integr Biol (2019) 23:70–85. doi: 10.1089/omi.2018.0206 30767728

[116] Wang Y Xie Q Tan H Liao M Zhu S Zheng L-L. Targeting cancer epigenetic pathways with small-molecule compounds: Therapeutic efficacy and combination therapies. Pharmacol Res (2021) 173:105702. doi: 10.1016/j.phrs.2021.105702 34102228

[117] Cho M-H Park J-H Choi H-J Park M-K Won H-Y Park Y-J. DOT1L cooperates with the c-Myc-p300 complex to epigenetically derepress CDH1 transcription factors in breast cancer progression. Nat Commun (2015) 6:7821. doi: 10.1038/ncomms8821 26199140 PMC4525167

[118] Zarković N Kalisnik T Loncarić I Borović S Mang S Kissel D. Comparison of the effects of Viscum album lectin ML-1 and fresh plant extract (Isorel) on the cell growth in *vitro* and tumorigenicity of melanoma B16F10. Cancer Biother Radiopharm (1998) 13:121–31. doi: 10.1089/cbr.1998.13.121 10850348

[119] Siegelin MD Schneider E Westhoff M-A Wirtz CR Karpel-Massler G. Current state and future perspective of drug repurposing in Malignant glioma. Semin Cancer Biol (2021) 68:92–104. doi: 10.1016/j.semcancer.2019.10.018 31734137

[120] Velicer CM Ulrich CM. Vitamin and mineral supplement use among US adults after cancer diagnosis: a systematic review. J Clin Oncol Off J Am Soc Clin Oncol (2008) 26:665–73. doi: 10.1200/JCO.2007.13.5905 18235127

[121] Wang J Li D Zhao B Kim J Sui G Shi J. Small molecule compounds of natural origin target cellular receptors to inhibit cancer development and progression. Int J Mol Sci (2022) 23:2672. doi: 10.3390/ijms23052672 35269825 PMC8911024

[122] Buyel JF. Plants as sources of natural and recombinant anti-cancer agents. Biotechnol Adv (2018) 36:506–20. doi: 10.1016/j.bioteChadv.2018.02.002 29408560

[123] Tsuji S Nakamura S Maoka T Yamada T Imai T Ohba T. Antitumour effects of astaxanthin and adonixanthin on glioblastoma. Mar Drugs (2020) 18:474. doi: 10.3390/md18090474 32962073 PMC7551886

[124] Oppermann H Faust H Yamanishi U Meixensberger J Gaunitz F. Carnosine inhibits glioblastoma growth independent from PI3K/Akt/mTOR signaling. PloS One (2019) 14:e0218972. doi: 10.1371/journal.pone.0218972 31247000 PMC6597087

[125] Hua D Zhao Q Yu Y Yu H Yu L Zhou X. Eucalyptal A inhibits glioma by rectifying oncogenic splicing of MYO1B mRNA *via* suppressing SRSF1 expression. Eur J Pharmacol (2021) 890:173669. doi: 10.1016/j.ejphar.2020.173669 33098832

[126] Shahcheraghi SH Lotfi M Soukhtanloo M Ghayour Mobarhan M Jaliani HZ Sadeghnia HR. Effects of galbanic acid on proliferation, migration, and apoptosis of glioblastoma cells through the PI3K/Akt/MTOR signaling pathway. Curr Mol Pharmacol (2021) 14:79–87. doi: 10.2174/1874467213666200512075507 32394847

[127] Martínez-Escardó L Alemany M Sánchez-Osuna M Sánchez-Chardi A Roig-Martínez M Suárez-García S. Gossypol treatment restores insufficient apoptotic function of DFF40/CAD in human glioblastoma cells. Cancers (2021) 13:5579. doi: 10.3390/cancers13215579 34771741 PMC8583586

[128] Yeh L-T Hsu L-S Chung Y-H Chen C-J. Tectorigenin inhibits glioblastoma proliferation by G0/G1 cell cycle arrest. Med Kaunas Lith (2020) 56:681. doi: 10.3390/medicina56120681 PMC7763962 33321738

[129] Tang Q Ren L Liu J Li W Zheng X Wang J. Withaferin A triggers G2/M arrest and intrinsic apoptosis in glioblastoma cells *via* ATF4-ATF3-CHOP axis. Cell Prolif (2020) 53:e12706. doi: 10.1111/cpr.12706 31642559 PMC6985693

[130] Maiti P Plemmons A Dunbar GL. Combination treatment of berberine and solid lipid curcumin particles increased cell death and inhibited PI3K/Akt/mTOR pathway of human cultured glioblastoma cells more effectively than did individual treatments. PloS One (2019) 14:e0225660. doi: 10.1371/journal.pone.0225660 31841506 PMC6913937

[131] Moskwa J Naliwajko SK Markiewicz-Żukowska R Gromkowska-Kępka KJ Nowakowski P Strawa JW. Chemical composition of Polish propolis and its antiproliferative effect in combination with Bacopa monnieri on glioblastoma cell lines. Sci Rep (2020) 10:21127. doi: 10.1038/s41598-020-78014-w 33273550 PMC7712839

[132] Khan M Ai M Du K Song J Wang B Lin J. Pyroptosis relates to tumor microenvironment remodeling and prognosis: A pan-cancer perspective. Front Immunol (2022) 13:1062225. doi: 10.3389/fimmu.2022.1062225 36605187 PMC9808401

[133] Mazumder A Cerella C Diederich M. Natural scaffolds in anticancer therapy and precision medicine. Biotechnol Adv (2018) 36:1563–85. doi: 10.1016/j.bioteChadv.2018.04.009 29729870

[134] Tamtaji OR Razavi ZS Razzaghi N Aschner M Barati E Mirzaei H. Quercetin and glioma: which signaling pathways are involved? Curr Mol Pharmacol (2022) 15:962–8. doi: 10.2174/1874467215666220211094136 35152872

[135] Zheng P Zhou C Ding Y Duan S. Disulfidptosis: a new target for metabolic cancer therapy. J Exp Clin Cancer Res (2023) 42:103. doi: 10.1186/s13046-023-02675-4 37101248 PMC10134647

[136] Zhang J Gao R Li J Yu K Bi K. Alloimperatorin activates apoptosis, ferroptosis, and oxeiptosis to inhibit the growth and invasion of breast cancer cells in vitro. Biochem Cell Biol (2022) 100:213–22. doi: 10.1139/bcb-2021-0399 35263194

[137] Abodief WT Dey P Al-Hattab O. Cell cannibalism in ductal carcinoma of breast. Cytopathol Off J Br Soc Clin Cytol (2006) 17:304–5. doi: 10.1111/j.1365-2303.2006.00326.x 16961662

